# Influence of Particle Size on the Mechanical Performance and Sintering Quality of Peanut Husk Powder/PES Composites Fabricated through Selective Laser Sintering

**DOI:** 10.3390/polym15193913

**Published:** 2023-09-28

**Authors:** Aboubaker I. B. Idriss, Chun-Mei Yang, Jian Li, Yanling Guo, Jiuqing Liu, Alaaeldin A. A. Abdelmagid, Gafer A. Ahmed, Hao Zhang

**Affiliations:** 1 College of Mechanical and Electrical Engineering, Northeast Forestry University, Harbin 150040, China; aboubakerbolad@outlook.com (A.I.B.I.); guo.yl@hotmail.com (Y.G.); nefujdljq@163.com (J.L.);; 2 Department of Mechanical Engineering, Faculty of Engineering Science, University of Nyala, Nyala P.O. Box 155, Sudan; 3 School of Civil Engineering, Southwest Jiaotong University, Chengdu 610031, China; alaa2016ch@gmail.com; 4 Department of Mechanical Engineering, College of Engineering, Sudan University of Science and Technology, Khartoum 11113, Sudan; gafergg89@hotmail.com

**Keywords:** selective laser sintering, PHP/PES (PHPC) composite, scanning electron microscopy inspection, mechanical test analysis, dimensional precision analysis, post-processing

## Abstract

This study intends to enhance the mechanical strength of wood–plastic composite selective laser sintering (SLS) parts by using a sustainable composite, peanut husk powder (PHP)/poly ether sulfone (PES) (PHPC). The study aims to address agricultural waste pollution by encouraging the eco-friendly utilization of such waste in SLS technology. To ensure the sintering quality and mechanical properties and prevent deformation and warping during sintering, the thermo-physical properties of PHP and PES powders were analyzed to determine a suitable preheating temperature for PHPC. Single-layer sintering tests were conducted to assess the formability of PHPC specimens with varying PHP particle sizes. The study showed the effects of different PHP particle sizes on the mechanical performance of PHPC parts. The evaluation covered various aspects of PHPC SLS parts, including mechanical strength, density, residual ash content, dimensional accuracy (DA), and surface roughness, with different PHP particle sizes. The mechanical analysis showed that PHPC parts made from PHP particles of ≤0.125 mm were the strongest. Specifically, the density bending strength, residual ash content, tensile, and impact strength were measured as 1.1825 g/cm^3^, 14.1 MPa, 1.2%, 6.076 MPa, and 2.12 kJ/cm^2^, respectively. Notably, these parameters showed significant improvement after the wax infiltration treatment. SEM was used to examine the PHP and PES powder particles, PHPC specimen microstructure, and PHPC SLS parts before and after the mechanical tests and waxing. Consequently, SEM analysis wholly confirmed the mechanical test results.

## 1. Introduction

Selective laser sintering (SLS, or LS) technology is an innovative additive manufacturing (AM) technique, first introduced by C. R. Deckard in 1988, which uses a CO_2_ laser beam to melt powder material within the SLS machine using three dimensions of part geometry data in the STL file [1,2,3,4]. In the technical process of SLS technology, the powder material particles dissolve and bond to create individual layers, progressively building a solid 3D object [5,6]. Biomass composite powders undergo a sintering process through SLS, where the nearby particles’ binder is melted and partially cured by the laser beam’s high temperature [6,7,8,9]. SLS technology offers several advantages over other AM methods: (1) cost-effective production of intricate geometric parts without requiring expensive tools, (2) reusability of non-sintered powder, and (3) increased stability and dimensional accuracy (DA) in the final SLS sintered parts compared to those produced via alternative technologies [10,11,12,13].

Current research on SLS technology focuses on ceramics, metals, polymers, and biomass composite materials, with applications in diverse industries, such as investment casting, medical equipment, furniture and equipment, automotive, construction, and aerospace [14,15,16,17,18,19,20,21]. The current materials are expensive, difficult to make, and some are not good quality, such as the biomass composite SLS parts. The conventional materials cannot meet the market demands, limiting the potential applications of SLS technology [10,17,22]. To address these limitations, there is an important need to explore and develop new and sustainable materials for SLS, considering factors such as the particle size, mixture ratios, and appropriate SLS processing parameters [23].

Eco-friendly biomass composite materials with relatively low melting temperatures are well suited for SLS, making it crucial to identify environmentally friendly SLS materials that can be produced with low energy consumption, low cost, and low CO_2_ emissions [18,24]. Li and colleagues, from China, have proposed using the waste of agricultural and forestry industries as a feedstock for SLS, showing promising results with materials such as sintered sisal fiber composite (SFPC), PCPC composite, wood composite, bamboo wood, eucalyptus wood, and rice husk–plastic composite powder [17,25,26,27,28,29]. However, different biomass sources yield powder materials with varying morphologies and particle sizes, directly impacting the laser’s forming performance and the quality of the final SLS parts [17,24,30,31,32]. Surprisingly, the previous studies did not report on the impact of the biomass powder particle size on the mechanical performance and forming quality of SLS parts.

Various complex shapes can be produced using SLS, but laser forming often leads to deformation and over-sintering in some of the resulting parts, mainly caused by inappropriate powder particle sizes [31]. Additionally, the interaction mechanism between powder materials and essential energy in the sintering process relies on the particle sizes of the raw materials [13,33,34]. As a result, the powder particle size significantly affects the DA, sintering quality, mechanical strength, and surface roughness of biomass SLS parts [13,22,23,25,29]. Hence, this paper aims to consider the effect of particle size on the mechanical strength, dimensional precision, surface roughness, and residual ash content of the manufactured SLS parts, considering the collective insights from prior research [13,22,23,25,29].

Peanut husk, similar to rice husk and wood powder, is an abundant agricultural waste that is often undervalued and underutilized. In countries such as Sudan, a major peanut husk producer, it is commonly burned for disposal, leading to environmental harm [24,29]. Peanut husk stands out because of its ease of crushing and milling, and it possesses a unique advantage of having a low melting point compared to other biomass composites [24]. Peanut husk powder is better than rice husk powder for making SLS parts because of its smooth and uniform spreading, which gives it high DA and great strength. The characteristic of peanut husk particles moving easily between PES powders is beneficial for making SLS parts of superior quality. Based on these helpful properties, PHP has been selected as the primary material incorporated into the PES matrix for the development of sustainable composite materials as feedstock for SLS. PES is a thermoplastic polymer that is stronger than many other types of plastics and works well at temperatures between 100 °C and 200 °C.

This study aims to examine the impact of the PHP particle size on mechanical properties and sintering quality, while proposing to repurpose peanut husk waste in SLS technology instead of disposing of it through burning. The PHPC is a sustainable material for SLS AM, with the potential to positively affect the environment by reducing carbon dioxide emissions from peanut husk burning. PHPC parts of varying particle sizes hold promise for applications in construction, and in manufacturing wooden floors, doors, and furniture, thereby reducing the demand for wood and its impact on forests. Moreover, the present PHPC composite is anticipated to offer economic advantages, as it becomes a sustainable material for SLS use, effectively addressing the material scarcity in SLS technology. Notably, parts manufactured using the optimized PHP particle size for PHPC demonstrate significant physical benefits, including remarkable mechanical strength, relatively low residual ash content, and high DA. These sintered parts are better than previous biomass–plastic composites, such as SFPC and PCPC, in terms of their mechanical properties.

A prior investigation on the peanut husk powder (PHP)/poly ether sulfone (PES) composite (PHPC) tackled challenges related to the powder mixture ratios and SLS parameters and effectively resolved them by determining the optimal mixture ratios and suitable SLS parameters [24]. However, there remains a knowledge gap regarding the impact of different peanut husk particle sizes on the mechanical performance and overall quality of resulting PHPC parts [24]. Therefore, optimizing the particle size range of peanut husk powder emerges as a critical factor in enhancing the production of high-quality SLS parts [17,25,27].

This paper presents a comprehensive study of different PHP particle sizes and PES powder, investigating their impact on the mechanical performance and forming quality of the resulting PHPC parts. Additionally, the sintering mechanism of PHPC with various PHP particle sizes was thoroughly analyzed, followed by assessments of surface quality, density, and mechanical performance. The primary focus of this study is to optimize the PHP particle size for enhanced sintering quality and mechanical performance, wherein sintering refers to the localized laser fusing and melting of PHPC matrix particles.

The study utilized different PHP particle sizes (0.149 mm, 0.125 mm, 0.105 mm, and 0.088 mm) mixed with PES in a ratio of 10%:90%. This study is one of the pioneering works investigating the impact of the PHP particle size on the mechanical performance, residual ash content, and sintering quality of the resulting PHPC SLS parts. To enhance the mechanical performance of the PHPC SLS parts, post-processing infiltration with pool wax and industrial paraffin was employed. The mechanical properties of PHPC SLS parts before and after post-processing were compared to highlight the beneficial effects of the post-processing method.

## 2. Materials and Methods

### 2.1. Raw Materials

**Figure 1:**
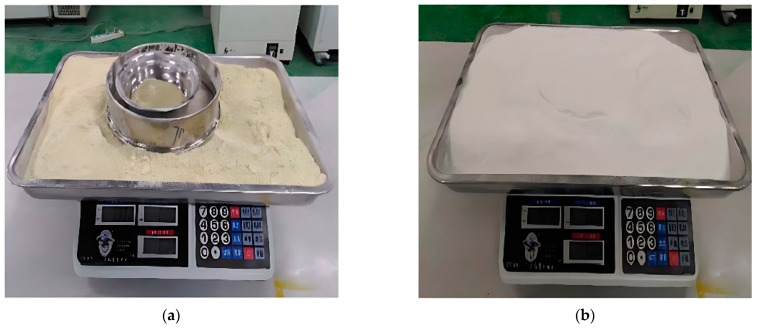
Raw materials: (**a**) pure PHP and (**b**) pure PES.

The main materials utilized here include PES (purchased from Shanghai TianNian Material Technology, Shanghai, China) and peanut husk (from South Darfur State, Sudan). The peanut husk was processed into PHP utilizing crushing machinery (Jiangsu Guibao Group Co., Ltd., Beijing, China). For different PHP particle sizes, an experimental design was applied, sifting the particles through 80, 100, 120, and 140 mesh screens using a vibrating sifter (Model ZS 350 Jiangsu Guibao Group Co., Ltd., Beijing, China) to eliminate agglomerated particles. The resulting PHP particle sizes were ≤0.149 mm, ≤0.125 mm, ≤0.105 mm, and ≤0.088 mm, respectively. After sifting, the PHP particles were dried at 100 °C for 3–4 h and then cooled to room temperature before being used in the PHPC composite. Photographs of pure PHP and PES are shown in Figure 1.

### 2.2. Preparation of PHPC

The PHP of various particle sizes and PES powders were blended in a specific volume proportion of 10%:90%. To achieve a uniform color, the mixture underwent mechanical mixing in a high-speed mixer. Afterward, the PHPC was thoroughly blended using a high-speed blender (SHR-10A, Zhang Jiagang Lanhang Machinery Co., Ltd., Suzhou, China) for 15 min until complete homogeneity was achieved. PES is a thermoplastic polymer known for its excellent thermal properties within the range of −100 °C to 200 °C.

**Figure 2:**
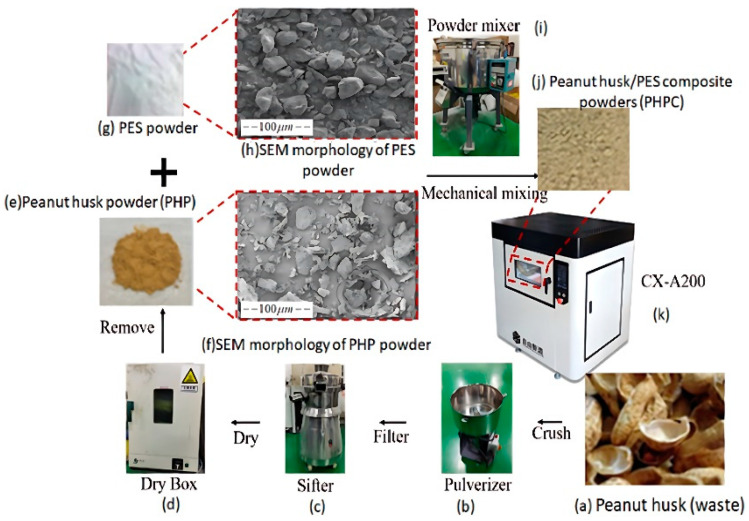
The preparation flow chart of the PHPC composite: (**a**) raw peanut husk material, (**b**) pulverizer machine for crushing, (**c**) sifter machine for sifting powders after crushing, (**d**) furnace device, (**e**) samples of pure PHP, (**f**) SEM morphology of pure PHP, (**g**) pure PES powders, (**h**) SEM morphology of pure PES powders, (**i**) powder mixing machine, (**j**) PHPC samples, and (**k**) 3d printing machine.

PHP tended to enhance the PHPC surface smoothness, flexibility, accuracy, and mechanical strength. The preparation flow chart of the PHPC composite is shown in Figure 2.

### 2.3. SLS Experiment

**Figure 3:**
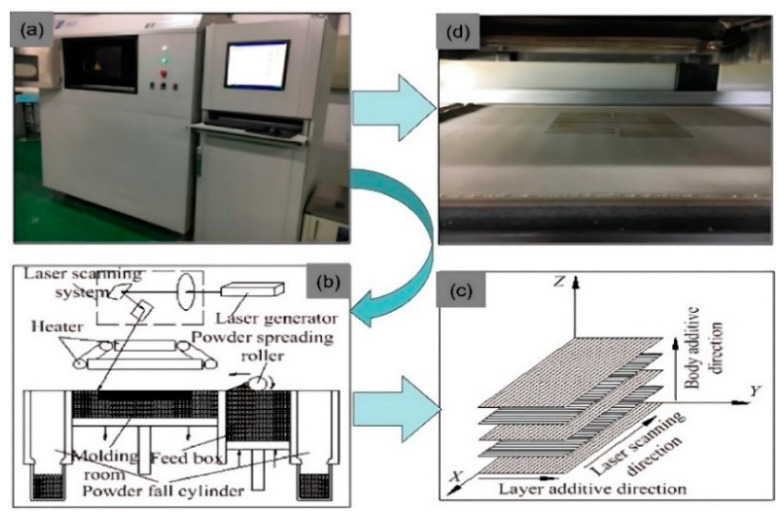
SLS building process using: (**a**) the AFS-360 SLS machine, (**b**) the SLS machine characteristic process, (**c**) laser scanning directions, and (**d**) samples under manufacturing in SLS.

PHPC samples were fabricated using the AFS-360 SLS machine (Beijing Longyuan Technology Ltd., Beijing, China). Figure 3 illustrates the process of AFS-360 SLS. SLS processing parameters were used, with an 82 °C preheating temperature, 14 W laser power, 0.15 mm layer thickness, 75 °C processing temperature, 2 m/s scan speed, and 0.2 mm scan spacing. To determine the optimal PHP particle size, four groups of PHPC specimens were prepared following the proposed experimental design. The SLS AFS-360 machine used a CO_2_ laser with a wavelength of 10.6 μm and a power of less than 55 W.

### 2.4. The Fabrication of the PHPC SLS Specimens

Tensile samples were designed in STL format following the GB/T1040-1992 standard [35], measuring 150 × 20 × 10 mm. Bending samples were also in STL format, prepared according to the GB/T9341-2008 standard [36], and measured 80 × 13 × 4 mm. For impact samples, the STL format adhered to the ISO179-2000 standard [37], with dimensions of 80 × 10 × 4 mm.

### 2.5. Dimensional Accuracy (DA)

The DA of different PHPC SLS parts was evaluated, targeting a nominal dimension of parts of 80 × 13 × 4 mm. The Vernier caliper was used to measure the dimensions. The analysis of DA resulted in identifying the best particle size for the PHP. The DA percentage of the produced PHPC parts was calculated using Equation (1):

(1) $$\delta \left(\%\right)=\left(1-\frac{\left[Lo-L\right]}{Lo}\right)$$

where *L* is the measured dimension (mm) of the printed part, $\delta \left(\%\right)$ is the DA (%), and Lo is the standard specified dimension (mm) of the printed part. The measured dimensions in the X, Y, and Z directions of the PHPC parts are illustrated in Table 1.

### 2.6. Mechanical Testing

**Figure 4:**
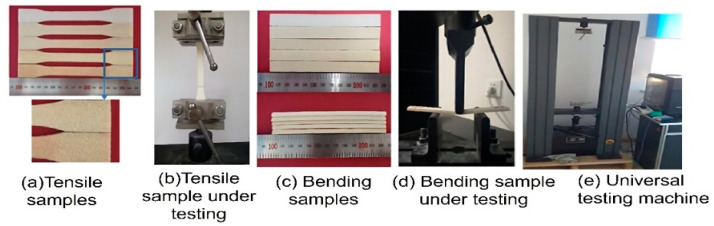
Photographs of the PHPC SLS specimens used for (**a**) the tensile testing, (**b**) a tensile specimen under testing, and (**c**) bending testing, showing (**d**) a bending specimen under testing, and (**e**) the universal testing machine.

The bending and tensile mechanical strengths of different PHPC SLS specimens were assessed using an electronic universal testing machine (Byes-3003, Shenzhen Sans Company, Shenzhen, China). Mechanical properties, including impact, bending, and tensile strength, were determined in triplicate for each part design, and the average values were reported based on the specified Chinese standard (GB/T1040-1992) [35]. Impact strength was measured in separate impact samples using a Charpy impact testing machine (Chengde, China) following the official Chinese standard (ISO 179-2000) [37]. Impact specimens had a span length of 64 mm, the velocity of the impact pendulum was 2.9 ${\mathrm{ms}}^{-1}$, and the impact energy was 2 J during testing. Statistical analyses were performed on the average mechanical values at a 95% confidence interval. Figure 4a–e presents the manufactured PHPC SLS specimens and the Byes-3003 machine used for testing.

### 2.7. Density

The density of PHPC SLS parts with different PHP particle sizes was determined by measuring their sizes and weights using a Vernier caliper and electronic balance, respectively. The density analysis was conducted on the specific dimensions of PHPC bending parts (80 × 13 × 4 mm) with different PCP particle sizes. The density of PHPC SLS specimens was determined using Equation (2):

(2) $$\rho =\frac{w}{l\times b\times h}$$

In the equation, *ρ* represents the density (g/cm^3^), *l* denotes the length (mm), *b* indicates the width (mm), *h* represents the thickness (mm), and *w* represents the mass of the SLS part (g).

### 2.8. Thermogravimetric Analysis (TGA) and Differential Scanning Calorimetry (DSC) Examinations

**Figure 5:**
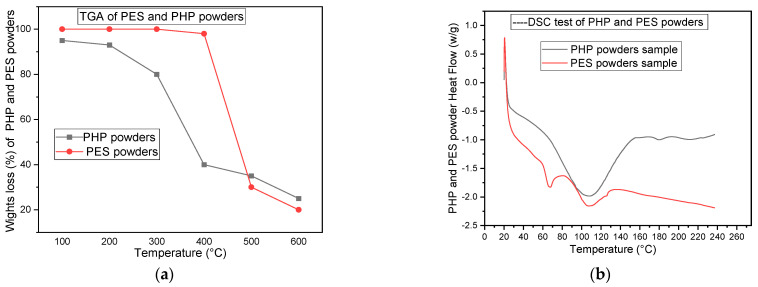
TGA and DSC curves of PHP and PES powders, displayed in (**a**) and (**b**), respectively.

The glass transition temperatures (Tg) of both PES and PHP were calculated using a differential scanning calorimetry (DSC) device. Based on the DSC experiment results, the optimal processing temperature for PHPC was determined. The DSC device (PerkinElmer, Waltham, MA, USA) was used to determine the Tg of pure PES powders and PHP. The measurements were recorded in °C for temperature and mg for weight. A sample weighing 3–5 mg was used for Tg testing. The testing temperature range was 20–240 °C, and the heating rate employed was 10 °C/min. For the thermogravimetric analysis (TGA) of PCP and PES powders, the PerkinElmer device test was employed. The test involved using a sample mass of 10 mg for both PCP and PES, and they were subjected to TGA with a heating rate of 10 °C/min within a temperature range of 100~600 °C. The TGA and DSC curves of PES and PHP can be seen in Figure 5a,b, respectively.

### 2.9. SEM Analysis

The FEI Quanta 200 SEM (Hewlett-Packard Company, Amsterdam, The Netherlands) was used to inspect the particle distribution and microstructure of pure PES powders, PHP, and PHPC specimens. Additionally, SEM was employed to observe the morphology of PHPC SLS parts manufactured with various PHP particle sizes before and after mechanical testing (breakage), as well as before and after post-processing treatment.

### 2.10. Surface Roughness Analysis

The surface roughness of PHPC SLS parts (80 mm × 13 mm × 4 mm) made with different PHP particle sizes was determined using a surface roughness tester.

### 2.11. Residual Ash Content Analysis

To determine the residual ash content in PHPC specimens, samples were burned using a box-type resistance furnace (SX2-512, Shanghai Shiyan Electric Furnace Co., Ltd., Shanghai, China). The experimental procedure involved placing crucibles containing PHPC samples into the furnace, heating them to 850 °C, and maintaining the temperature for 2 h. After the heating and burning phase, the electric furnace was turned off, and the PHPC samples were removed and weighed at room temperature. The residual ash content of the burned PHPC parts manufactured with various PHP particle sizes was then calculated using Equation (3):

(3) $$f=\frac{a-b}{c}\times 100$$

In the equation, *f* represents the residual ash content value (%) of the PHPC samples, *a* denotes the mass of the crucible containing residual ash (g), *b* is the mass of the crucible (g), and *c* is the mass of the sample before burning (g).

### 2.12. Post-Processing Treatment

**Figure 6:**
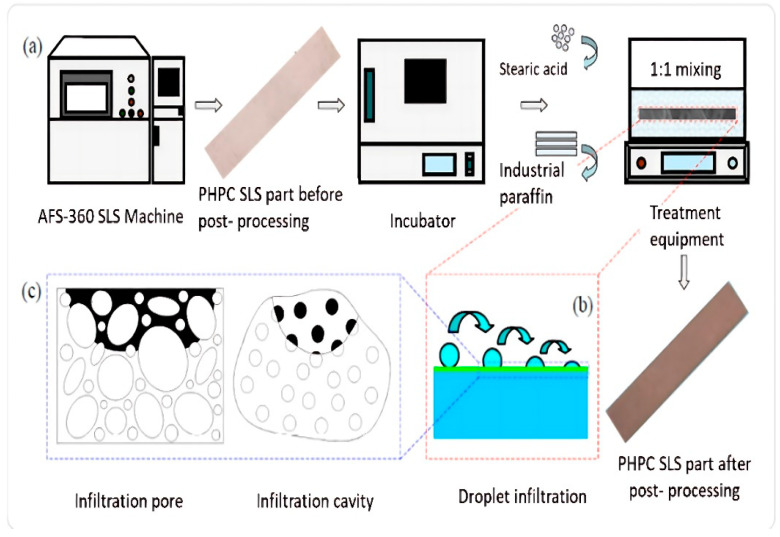
The flow chart of the post-processing steps, depicting the parts before and after post-processing, as follows: (**a**) SLS machine, (**b**) droplet infiltration, and (**c**) visualization of the infiltration pore.

The SLS PHPC parts exhibited satisfactory mechanical strength, including bending, tensile, and impact strength. However, they often had a low porous surface and denser holes. To improve these aspects, post-processing methods, particularly wax infiltration, were implemented following the flow chart shown in Figure 6. Initially, the PHPC parts were carefully removed from the AFS-360 SLS machine, cleaned, and then insulated in an electrical heating thermostat at 70 °C for 2 h. Subsequently, the specific PHPC parts were slowly immersed in a wax pool, allowing wax to infiltrate the holes through capillarity. This action improved the quality and strength of the PHPC parts by filling the gaps between particles. The wax infiltration caused a small increase in the size of the PHPC SLS specimens, which slightly affected their accuracy. The DA of waxed PHPC SLS parts can be enhanced by using a polishing method and an electric vacuum cleaner to remove adhesion on sintered waxed parts.

## 3. Results and Discussion

### 3.1. Single-Layer Sintering Analysis

Analyzing the particle size is important for the SLS process of PHP. It impacts the spread of powders and the quality of PHPC SLS specimens. Since PHP particles are mechanically crushed, their shapes are non-uniform, making it essential to investigate how the particle size and shape affect the powder spread distribution. Single-layer sintering tests were conducted with different PHP particle sizes, resulting in varying particle shape distributions, which directly influenced the surface quality of the PHPC SLS parts.

**Figure 7:**
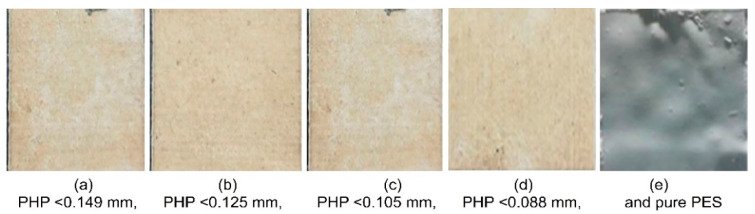
Single-layer sintered PHPC samples with various PHP particle sizes: (**a**) PHP ≤ 0.149 mm, (**b**) PHP ≤ 0.125 mm, (**c**) PHP ≤ 0.105 mm, (**d**) PHP ≤ 0.088 mm, and (**e**) pure PES.

Prior to SLS testing for PHPC, each design was subjected to a single-layer sintering experiment to assess and validate the feasibility of utilizing PHPC in SLS, and thereafter, to determine the optimal PHP particle size. The tests showed that PHPC is highly formable with strong adhesion and an excellent interface neck between PES and PHP particles (as seen in the Microstructure Analysis Section and Figure 7). Mechanical tests were then conducted on various PHPC SLS parts with different PHP particle sizes (≤0.149, ≤0.125, ≤0.105, and ≤0.088 mm). The single-layer sintering tests showed that all PHP particle sizes in this study were feasible for SLS. However, the best sintering qualities were achieved with PHP particle sizes within the range of ≤0.125 mm, outperforming other sizes and the pure PES powder.

### 3.2. Selective Laser Sintering Test Experiment

**Figure 8:**
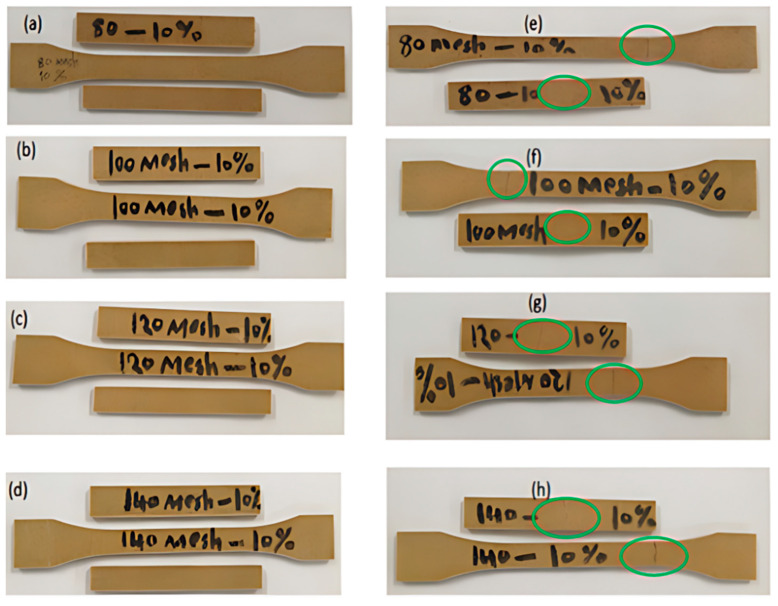
PHPC SLS samples with different PHP particle sizes (≤0.149 mm, ≤0.125 mm, ≤0.105 mm, and ≤0.088 mm), respectively, before and after mechanical testing: (**a**–**d**) PHPC SLS parts subjected to bending, tensile, and impact tests before mechanical testing, and (**e**–**h**) the samples after breakage during mechanical testing.

The ASF-360 SLS process yielded PES and PHPC parts with high forming quality and smooth brown surfaces (Figure 3a,b). The produced PHPC parts showed good mechanical strength and dimensional accuracy, making this approach suitable for AM technology in manufacturing construction components, furniture, and wooden floors. Figure 8 depicts photographs of the bending, tensile, and impact tests on the PHPC SLS samples manufactured with various PHP particle sizes before and after mechanical testing.

### 3.3. Mechanical Properties’ Analysis

**Figure 9:**
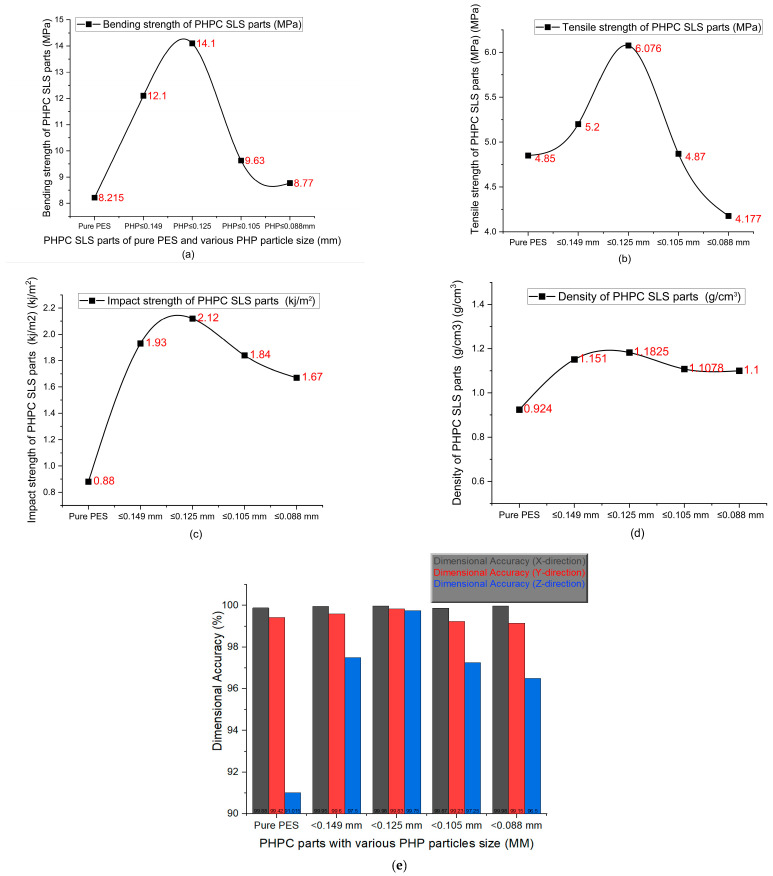
Mechanical characteristics of the SLS PHPC parts with different PHP particle sizes, including (**a**) bending strength, (**b**) tensile strength, (**c**) impact strength, (**d**) density, and (**e**) DA in the x, y, and z directions.

Figure 9 shows the mechanical strength, density, and surface roughness of the PHPC SLS parts. These parameters are essential for evaluating the SLS product performance and forming quality. The mechanical performances of pure PES and PHPC SLS parts manufactured with various PHP particle sizes were obtained. The influence of different sizes of PHP particles on the bending strength, density, tensile strength, DA, impact strength, residual ash content, and surface roughness was meticulously analyzed. The data in Figure 9 reveal that decreasing the PHP particle size initially resulted in an increased mechanical strength (both bending and tensile strengths), density, and impact strength of the PHPC SLS parts. The highest values, of 14.1 MPa and 6.076 MPa for bending and tensile strengths, respectively, 1.1825 g/cm^3^ for density, and 2.12 kJ/cm^2^ for impact strength, were achieved with a PHP particle size of ≤0.125 mm.

**Figure 10:**
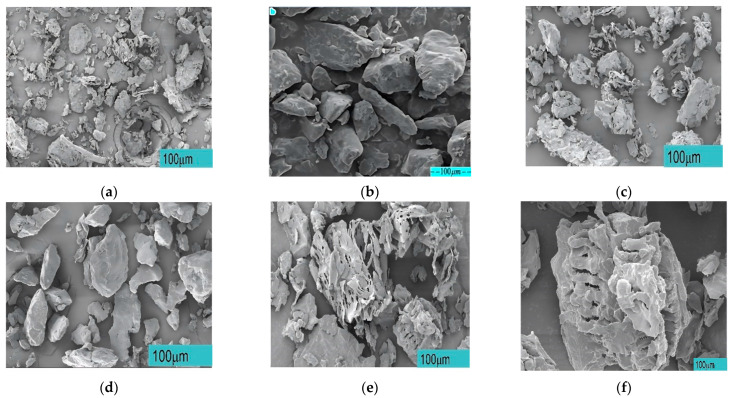
Surface morphology of different samples: (**a**) pure peanut husk powder (PHP) sample, (**b**) pure PES sample, (**c**) PHPC parts with PHP particle size ≤0.149 mm, (**d**) PHPC parts with PHP particle size ≤0.125 mm, (**e**) PHPC parts with PHP particle size ≤0.105 mm, and (**f**) PHPC parts with PHP particle size ≤0.088 mm.

**Figure 11:**
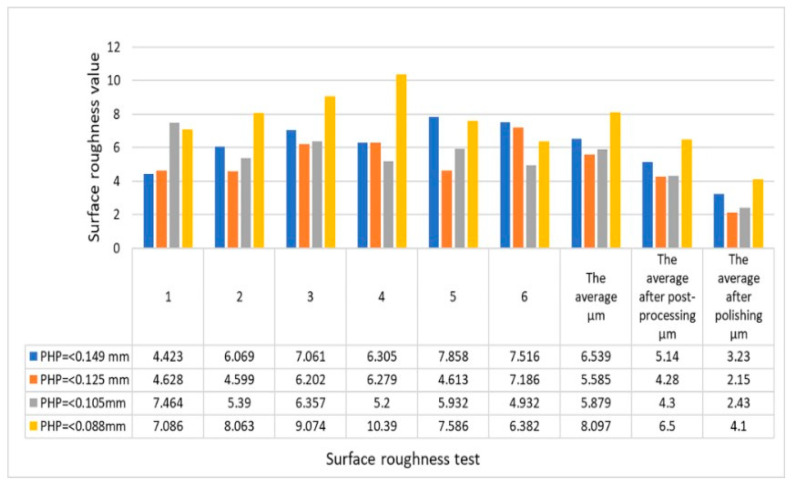
Surface roughness of the PHPC SLS parts made from various PHP particle sizes before and after post-processing (wax infiltration and polishing).

**Figure 12:**
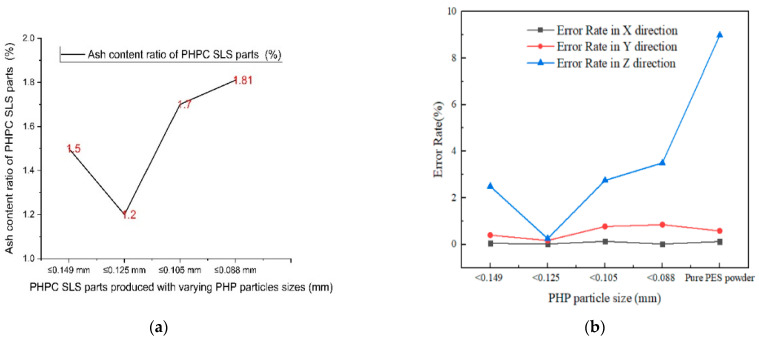
The ash content (**a**) and error rate (**b**) of the PHPC SLS parts produced using different PHP particle sizes.

Further analysis in Figure 9 shows that the mechanical properties of the PHPC SLS specimens initially improved with the decreasing PHP particle size (ranging from ≤0.149 to ≤0.125 mm) and then rapidly reduced. Identifying the suitable PHP particle size enhanced the forming quality and mechanical strength of the PHPC SLS specimens during sintering via promoting full mixing and complete melting of the PHPC samples (Figure 10). This led to strong cohesion between the PES and PHP powder particles, improving the surface roughness quality of the PHPC SLS parts (Figure 11). Additionally, the DA, density, mechanical strength (Figure 9), and residual ash content (Figure 12a) were enhanced.

In the SLS process, pure PES powder tends to over-decompose, resulting in a reduced sintering quality of the produced pure PES SLS parts (Figure 7e), especially in the center where high laser energy is concentrated. However, the addition of PHP to PES powder helps absorb a significant portion of the laser energy, reducing this damage (Figure 7a–d and Figure 10c–f). Through mechanical testing, the optimal PHP particle size for PHPC SLS parts was found to be ≤0.125 mm, as it exhibited the best mechanical properties, including bending, tensile, and impact strength, as well as density and residual ash content (Figure 9 and Figure 12a). This sintered part also showed excellent DA and smooth surface roughness, outperforming pure PES and other PHP particle sizes (Figure 9e and Figure 11).

Excessively small PHP particle sizes (PHP ≤ 0.088 mm) resulted in a reduced density and mechanical strength, along with an increased residual ash content. This was due to insufficient bonding between the PES binder and the very small PHP particles, leading to internal holes and a decreased mechanical strength, density, and DA of the PHPC SLS parts (Figure 10e,f). The reduction in the PHP particle size from ≤0.125 to ≤0.105 mm significantly decreased the density and mechanical strengths of the PHPC parts (Figure 9a–e) due to weakened sintering necks between PES and PHP particles (Figure 10d,e). Continuously reducing the PHP particle size further weakened the link efficiency in the sintered parts (Figure 10e,f), resulting in extensively reduced mechanical properties (Figure 9) and an increased residual ash content (Figure 12a). Consequently, the PHP particle size ≤ 0.125 mm is recommended as the optimal size in this work, considering its superior mechanical properties and lower error rate in all directions (Figure 12b); thus, for future applications of PHPC, the PHP particle size ≤ 0.125 mm is advised because of its favorable properties.

**Figure 13:**
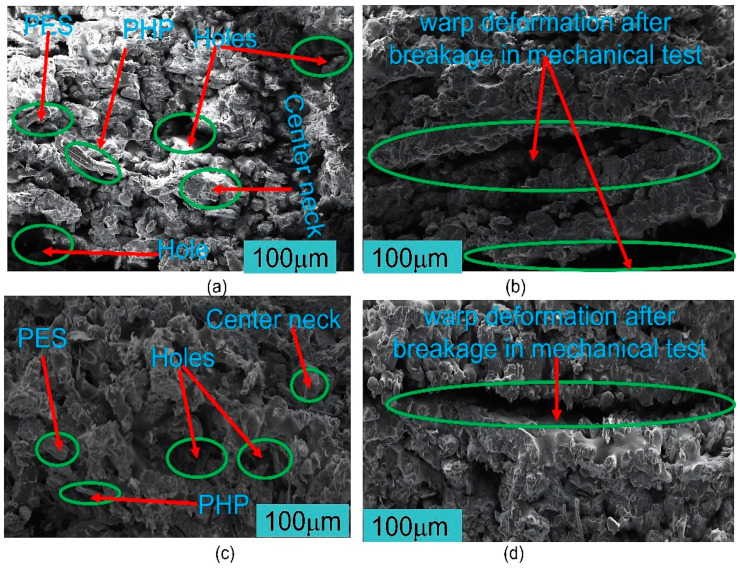

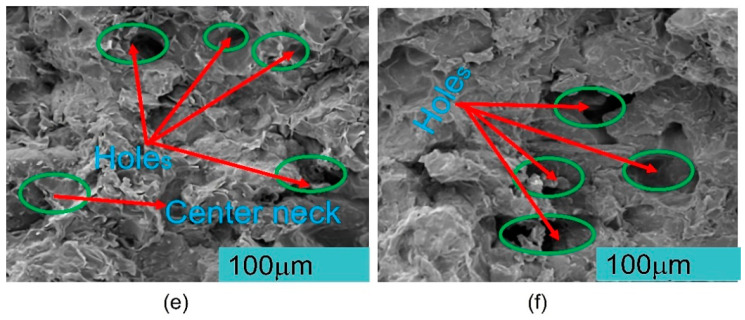
SEM images of the PHPC SLS parts with PHP ≤ 0.149 mm: (**a**) before and (**b**) after the mechanical experiment, as well as the PHPC SLS parts with PHP ≤ 0.125 mm: (**c**) before and (**d**) after the mechanical experiment. PHPC SLS specimens with PHP ≤ 0.149 mm (**e**) after the waxing process, and the PHPC SLS part with PHP ≤ 0.125 mm (**f**) after the waxing process.

Post-processing was implemented to enhance the density, surface roughness, and mechanical strength of the PHPC SLS specimens. Practically, wax infiltration filled most of the interior pores (Figure 13e,f), leading to an improved mechanical strength (tensile, bending, and impact), density, and surface roughness for the PHPC SLS parts (≤0.125 mm), with values increased to 7.476 MPa, 15.7 MPa, 2.96 KJ/cm^2^, 2.0625 g/cm^3^, and 3.58 μm, respectively. However, the post-processing slightly increased the dimensions of the PHPC SLS parts, shown to negatively affect the DA.

Through single-layer sintering experiments, SEM observation, and mechanical tests, it was found that PHPC parts with PHP ≤ 0.125 mm exhibited the best density, DA, mechanical strength, surface quality, and minimal residual ash content (Figure 7, Figure 9, Figure 10 and Figure 12a).

Comparing the SEM micrographs, Figure 13a–d illustrated the differences between the PHPC SLS parts produced from PHP ≤ 0.125 mm and those from PHP ≤ 0.149 mm, before and after breakage in the mechanical tests. Before breakage, the PHP ≤ 0.125 mm SLS specimen showed a higher density and a more extensive sintering neck than the PHP ≤ 0.149 mm specimen (Figure 13c and a, respectively). After breakage, the PHP ≤ 0.125 mm specimen exhibited warp deformation, specifically at the point of breakage (Figure 13d), while the PHP ≤ 0.149 mm specimen displayed warp deformation covering a larger area (Figure 13b). PHP specimens of ≤0.125 mm exhibited good density, a high impact strength, and fewer holes, making it a suitable choice for PHPC applications.

### 3.4. Statistical Analyses

**Figure 14:**
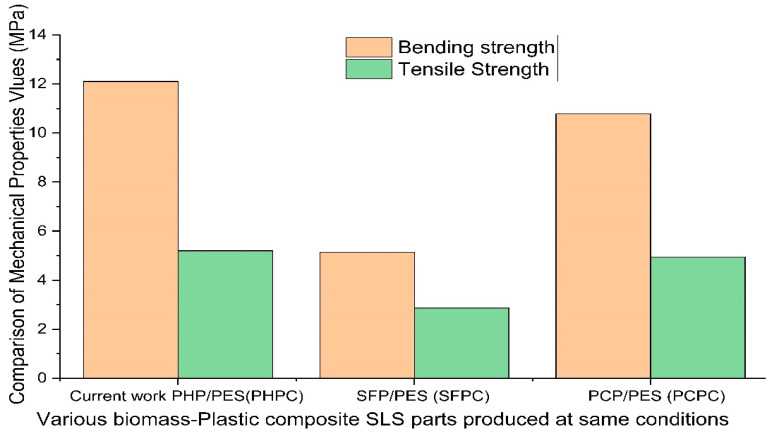
A comparison of the mechanical properties of SLS biomass–plastic composites fabricated using identical particle sizes of biomass powders and SLS parameter conditions.

The experimental results were verified for reliability by using the mean value and 95% confidence interval for statistical analysis. The repeatability experiment results are provided in Table 2, showing the lower and upper limits of the mean (tests) as well as the standard deviation (SD) of the mean. Statistical analyses revealed that overall, the measured data for the tensile and bending strengths fell within the 95% confidence interval, confirming the reliability of the test experiments. The comparison in Figure 14 shows that the mechanical properties of PHPC were superior to those of previously reported wood composite materials, such as SFP/PES (SFPC) and PCP/PES (PCPC) composite materials [11,15]. The related, determined tensile force loading (N) for the bending and tensile strengths (MPa) is clearly shown in Table 3.

### 3.5. SEM Microstructure Analysis

The formability and mechanical performance of PHPC SLS parts are influenced by the interfacial bonding mechanism and formability between PHP and the amorphous PES matrix, as well as the spreading of powder particles. Additionally, laser power serves as a key input for the combined PHPC biomass composite powders, with the particle size of PHP biomass powders also affecting the powder absorption. Figure 10 displays SEM photographs of fracture surfaces for various PHPC specimens prepared with different PHP particle sizes (≤0.149, ≤0.125, ≤0.105, and ≤0.088 mm), pure PHP, and pure PES. It is clear from Figure 10a that the pure PHP specimen exhibits an irregular surface with rough and nonuniform particles. In contrast, Figure 10b shows that the surface of PES powders is smoother and flatter than PHP, showing better fitting and matching for a good sintering neck between these powders. During mixing, the pure PHP is coated with PES powders, and some PHP fibers act as reinforcement to improve the mechanical properties. The rate of improvement varies depending on the particle size of the PHP in the PHPC composite.

The PHP sample with a particle size of ≤0.125 mm displayed uniform distribution of PES and PHP particles without noticeable agglomeration (Figure 10d). The bonding interface and sintering neck between PHP and PES were superior compared to PHP samples with particle sizes of ≤0.149 mm, ≤0.105 mm, and ≤0.088 mm, as well as pure PES samples (Figure 10c,e,f). An appropriate PHP particle size in PHPC resulted in enhanced mechanical properties, such as density, surface quality, DA, and mechanical strengths, in the sintered part, as well as an improved sintering quality and stability of pure PES SLS. Notably, in Figure 10d (PHP ≤ 0.125 mm), a major portion of PHP powder was coated with the PES matrix, leading to a lower porosity and fewer holes compared to other PHP particle sizes, and outperforming the pure PES specimen. The effective bonding and wrapping between the optimal PHP particles size (≤0.125 mm) in PHPC generated multiple continuous sintering zones (Figure 10d), allowing PES to efficiently absorb laser radiation, melt, and improve the liquidity of PES powders and increase the bonding.

Conversely, with smaller PHP particle sizes, a significant portion of the PHP particles were only partially coated with the PES matrix, while some adhered and dispersed within the PES matrix (Figure 10e–f). Consequently, the PES powders often failed to absorb sufficient power, resulting in a decreased liquidity of the PES powders and a reduced forming quality of the PHPC sintered parts. This led to a lower mechanical strength and density of the PHPC SLS parts of the PHP specimens of ≤0.105 mm and ≤0.088 mm, compared to the ≤0.125 mm and ≤0.149 mm specimens, as evident in Figure 9. Additionally, PHPC parts of PHP ≤ 0.105 and ≤0.088 mm specimens exhibited a lower surface quality than others, as depicted in Figure 11. Furthermore, these specimens displayed a higher residual ash content in the testing (Figure 12). Despite the pure PES specimen (Figure 10b) having relatively few holes and a dense microstructure, the PHP ≤ 0.125 mm specimen demonstrated improved surface roughness, density, and mechanical strength compared to the neat PES.

SEM examination was performed on PHPC SLS samples both before and after mechanical testing to assess deformation resulting from the breakage during the mechanical test (Figure 13a–d), which was discussed earlier in Section 3.3. Additionally, SEM analysis was carried out on the cross-section morphologies of PHPC SLS samples with PHP ≤ 0.149 mm and PHP ≤ 0.125 mm, before and after treatment via wax infiltration (Figure 13e,f), to show the positive impact of the wax infiltration method. Therefore, the PHPC SLS part with PHP ≤ 0.125 mm exhibited a relatively higher density and smaller internal holes compared to the part with PHP ≤ 0.149 mm (Figure 13a,c). Following the wax treatment, the size and quantity of internal holes in the PHP ≤ 0.125 mm part slightly reduced, and the bonding interface became relatively loose (Figure 13f) in comparison to the waxed parts of PHP ≤ 0.149 mm and PHP ≤ 0.088 mm (Figure 13e). The primary reason behind this observation is that in the case of PHP ≤ 0.125 mm, the internal structure of the PHPC SLS part is dense, making it difficult for liquid wax to permeate inside and fill the internal holes. For larger and smaller particle sizes (PHP ≤ 0.149 mm and PHP ≤ 0.088 mm), the internal structure of the PHPC SLS parts is relatively loose, facilitating easy permeation of the liquid wax inside. Consequently, excellent waxed parts of PHPC were obtained for parts with weak and low density (PHP ≤ 0.088 mm and PHP ≤ 0.149 mm), respectively. As a result, the quantity rate of internal pores in the waxed (PHP ≤ 0.125 mm) part was higher than parts of PHP ≤ 0.149 mm, and the sintering neck was small (Figure 13e,f). From the SEM analysis, it was determined that the PHPC part with PHP ≤ 0.125 mm had fewer pores and denser structures in PHPC SLS. However, the wax treatment had a weaker influence due to the low liquid wax permeability inside the holes of PHPC SLS. Consequently, the wax treatment had a more significant impact on the PHP ≤ 0.149 mm part compared to the PHP ≤ 0.125 mm part.

### 3.6. Sintering Temperature Analysis of the PHPC

SLS technology mainly relies on thermal effects. Since peanut husk powder (PHP) biomass lacks a melting point, amorphous thermoplastic powder (PES) plays a crucial role in forming PHPC parts. The Tg values of PHP and PES were calculated via DSC, allowing the estimation of preheating temperatures for the PHPC composite. To prevent warping and decomposition during sintering, the PHPC powders were heated within a limited temperature range (sintering window) based on the DSC assessment results. The sintering window was defined between the softening point (Ts) and the caking temperature (Tc) obtained from the DSC curve.

Here, PES is a non-crystallizable polymer powder, and its softening point corresponds to the Tg of PHP and PES powders. However, the caking temperature (Tc) could not be calculated from the DSC curves and was estimated directly during SLS manufacturing. Prior to the DSC experiment, the thermal decomposition temperatures (TGA) of the principal components of PHPC (PHP and PES) were calculated individually (Figure 6a). The TGA test revealed that the starting decomposition temperatures of the PES powder and PHP were 408 °C and 298 °C, respectively. To ensure that the maximum temperature of the PHPC composite in the DSC experiment remained below 298 °C, the DSC test temperature was chosen as a maximum of 240 °C.

DSC curves of PES and PHP samples are depicted in Figure 6b. Pure PES powder exhibited a Tg (Ts) value of 66 °C, while pure PHP had a Tg of 70 °C. Complete caking occurred at 95 °C for PES and 106 °C for PHP. These temperatures define the sintering window, and to ensure good forming quality and high mechanical strengths of PHPC SLS parts, the PHP and PES powder specimens should be preheated within specific temperature ranges of 66 °C to 95 °C for PES and 70 °C to 106 °C for PHP, respectively. To verify the sintering window condition and validate the thermal test design, all PHPC specimens were preheated to 82 °C during SLS processing, which falls within the suitable range.

## 4. Conclusions

The PHPC composite, composed of PES and PHP, was designed to create a biomass composite material to address material shortages and reduce costs in SLS materials. This study aimed to improve the mechanical strength of PHPC SLS parts by finding the ideal PHP particle size and using post-processing techniques. The results of the study revealed several noteworthy findings, which have shown that:

- PHPC SLS parts achieved superior mechanical performance with PHP particles ≤0.125 mm.
- Reducing the PHP particle size initially improved the strength, density, DA, and surface roughness of PHPC SLS parts, along with the residual ash content. However, further reduction decreased these properties.
- PHPC SLS specimens had minimal quantities and inner hole sizes with PHP particles ≤0.125 mm, but these increased at ≤0.088 mm.
- PHP particles ≤0.125 mm had the highest impact strength (2.12 kJ/cm^2^), tensile strength (6.076 MPa), density (1.1825 g/cm^3^), and bending strength (14.1 MPa). These attributes decreased as the PHP particle size decreased from ≤0.105 mm to ≤0.088 mm. A better DA in the X, Y, and Z directions was also shown compared to pure PES SLS parts, as well as a lower residual ash content and favorable surface roughness, compared to the other parts.
- The SEM photos showed that PHP ≤ 0.125 mm is the best particle size for PHPC SLS because of the largest bonding area and the small pores observed.
- The mechanical strength and surface roughness of PHPC SLS were enhanced by post-processing with wax infiltration and polishing. Density, bending strength, impact strength, and tensile strength increased to 2.0625 g/cm^3^, 15.7 MPa, 2.96 kJ/cm^2^, and 7.476 MPa, respectively. Surface roughness improved from 5.585 μm to 3.58 μm due to wax infiltration into the holes, with a slight decrease and subsequent improvement in DA after polishing.
- Optimized PHPC SLS parts had superior mechanical properties compared to other biomass composites, making them suitable for applications in AM technology, such as the furniture industry and for various wooden flooring options.
- Incorporating PHP into the PHPC composite not only enhances the SLS manufacturing sustainability but also produces strong, eco-friendly parts, making it promising for wider industrial use.

## Figures and Tables

**Table 1:** The specific measured dimensions of the PHPC produced parts (10/90) with various PHP particle size ranges.

PHPC SLS Parts	Width (mm)	Thickness (mm)	Length (mm)
PHP ≤ 0.149 mm	13.052	4.1	80.04
PHP ≤ 0.125 mm	13.1	4.01	80.01
PHP ≤ 0.105 mm	13.1	4.11	79.9
PHP ≤ 0.088 mm	13.12	4.14	80.014

**Table 2:** The repeatability of mechanical property experiments for SLS specimens manufactured from PHPC with different PHP particle sizes, along with the corresponding 95% confidence intervals for the mean and standard deviation (SD).

Parts at 10/90	Bending Strength Test Repeatability			Mean	95% Confidence Intervals for the Means	Tensile Strength Test Repeatability			Mean	95% Confidence Intervals for the Means	Standard Deviation (SD)	
	Test 1	Test 2	Test 3			Test 1	Test 2	Test 3			Bending	Tensile
PES	8.15	8.28	8.23	8.215	Lower = 7.22 Upper = 9.22	4.83	4.9	4.81	4.85	Lower = 4.27 Upper = 5.43	0.065574385	0.047258156
≤0.149 mm	12.6	11.9	11.8	12.1	Lower = 11.52 Upper = 12.68	5.1	5.15	5.36	5.2	Lower = 4.62 Upper = 5.78	0.34588989	0.13796135
≤0.125 mm	14	14.1	14.2	14.1	Lower = 13.52 Upper = 14.68	5.93	6.1	6.2	6.076	Lower = 5.49 Upper = 6.66	0.1	0.13650397
≤0.105 mm	9.8	9.9	9.2	9.63	Lower = 9.05 Upper = 10.21	4.8	4.8	5	4.87	Lower = 4.29 Upper = 5.45	0.37859389	0.11547005
≤0.088 mm	9.4	8.8	8.3	8.77	Lower = 8.19 Upper = 9.4	4.3	4.06	4.17	4.177	Lower = 3.6 Upper = 4.76	0.55075705	0.12013881

**Table 3:** The related, determined tensile force loading (N) for the bending and tensile strengths (MPa).

SLS Parts	Bending Strength (MPa)	The Related Tensile Force Loading (N)	Tensile Strength (MPa)	The Related Tensile Force Loading (N)
PES	8.215	138	4.85	102
≤0.149 mm	12.1	152.5	5.2	109
≤0.125 mm	14.1	158	6.076	115.7
≤0.105 mm	9.63	146	4.87	102.6
≤0.088 mm	8.77	140	4.177	99.8

## Data Availability

Not applicable.
